# Editorial Comment: Proximal Urethrostomy Versus Urethroplasty for Complex Urethral Strictures

**DOI:** 10.1590/S1677-5538.IBJU.2024.9912

**Published:** 2024-05-20

**Authors:** Luciano A. Favorito

**Affiliations:** 1 Unidade de Pesquisa Urogenital Universidade do Estado do Rio de Janeiro Rio de Janeiro RJ Brasil Unidade de Pesquisa Urogenital - Universidade do Estado do Rio de Janeiro - Uerj, Rio de Janeiro, RJ, Brasil;; 2 Serviço de Urologia Hospital Federal da Lagoa Rio de Janeiro RJ Brasil Serviço de Urologia, Hospital Federal da Lagoa, Rio de Janeiro, RJ, Brasil

Nir J Rahav ^1^, Mohamad Udah ^2^, Sarit Cohen ^2^, Tali Bdolah-Abram ^3^, Boris Chertin ^2^, Ofer Z. Shenfeld ^1^

^1^ Center for Reconstructive and Functional Urology, Shaare Zedek Medical Center, Jerusalem, Israel; ^2^ Department of Urology, Shaare Zedek Medical Center, Jerusalem, Israel; ^3^ The Hebrew University Medical School, Jerusalem, Israel

Eur Urol Open Sci. 2024 Mar 6:62:91-98.

DOI: 10.1016/j.euros.2024.02.008 | ACCESS: 38486615

## COMMENT

Complex anterior urethral stricture (CUS) are difficult to surgical management. Several options are used to treat this condition. Some options are done in stages and with a significant chance of complications. In present paper Rahav and collegues from Israel (1) shows a interesting study about the options to treat the complex urethral strictures. The objective of the paper was to compare proximal urethrostomy and urethroplasty for CUS using objective measures and validated questionnaires, and to evaluate trends in subgroups of patients who underwent proximal urethrostomy as the intended defini- tive treatment versus first-stage urethroplasty.

The authors in a retrospective study from 2004 to 2021compared 57 proximal urethrostomy and 75 urethroplasty patients for CUS (strictures >6 cm, recurrent pos- turethroplasty strictures, or CUS due to lichen sclerosus or past hypospadias surgery). The primary outcome was a recurrent stricture at a minimal follow-up of 1 yr. The secondary outcomes included vali- dated questionnaires, uroflowmetry, and residual urine volume. Comparisons of the two groups revealed no significant differences in stricture recurrence, results of validated questionnaires, or objective measures of urination. The authors concluded that proximal urethrostomy is equivalent to urethral reconstruction, and it should be offered as a viable solution for complex urethral stricture. This option is very useful, easy to do and the majority of the patients has a great satisfaction.
